# Nodal Marginal Zone Lymphoma with Simultaneous Transformation at Diagnosis and Pleural Effusion as the Initial Presentation: A Case Report

**DOI:** 10.15388/Amed.2025.32.1.21

**Published:** 2025-02-18

**Authors:** Konstantinos Dodos, Vasileia Tsampika Kalamara, Vasiliki Epameinondas Georgakopoulou, Paraskevi Kavoura

**Affiliations:** 1 Laboratory of Physiology, School of Medicine, Aristotle University of Thessaloniki, Thessaloniki, Greece; 2 Department of Pathophysiology, Laiko General Hospital, National and Kapodistrian University of Athens, Athens, Greece; 3 Department of Respiratory Medicine, General Oncological Hospital of Kifissia “Agioi Anargyroi’’, Athens, Greece

**Keywords:** B-cell non-Hodgkin lymphoma, histologic transformation, pleural effusion, diffuse large B-cell lymphoma, nodal marginal zone lymphoma, B ląstelių ne Hodžkino limfoma, histologinė transformacija, pleuros efuzija, difuzinė didelių B ląstelių limfoma, mazgo ribinės zonos limfoma

## Abstract

Nodal Marginal Zone Lymphoma (NMZL) is a rare, indolent subtype of B-cell non-Hodgkin lymphoma. This case report presents a 67-year-old female diagnosed with NMZL, which had transformed into Diffuse Large B-cell Lymphoma (DLBCL) at the time of diagnosis. The patient’s initial presentation involved a dry cough and pleural effusion, a symptom rarely reported in NMZL cases. Diagnostic procedures, including cytology and immunohistochemistry, confirmed the presence of monoclonal B lymphocytes and identified markers consistent with NMZL transformation to DLBCL. The findings highlight the diagnostic challenges associated with NMZL due to the lack of specific immunohistochemical markers, emphasizing the need for histopathological analysis to distinguish NMZL from other lymphomas, such as follicular lymphoma. This case underscores the importance of early detection and differentiation in lymphomas presenting with pleural effusion, as transformation to aggressive forms like DLBCL significantly impacts the prognosis and treatment approaches. Despite the rarity of transformation at diagnosis, clinicians must consider it in NMZL cases with atypical presentations.

## Abstract (it)

Mazginė marginalinė zonos limfoma (NMZL) yra reta, lėtai progresuojanti B ląstelių ne Hodžkino limfomos porūšis. Šiame klinikiniame pranešime aprašoma 67 metų moteris, kuriai buvo diagnozuota NMZL, jau transformavusis į difuzinę didelių B ląstelių limfomą (DLBCL) diagnozės metu. Pacientė kreipėsi dėl sauso kosulio ir pleuros išsiliejimo – simptomo, kuris NMZL atvejais pasitaiko retai. Atliekant diagnostinius tyrimus, įskaitant citologiją ir imunohistochemiją, buvo nustatytas monokloninių B limfocitų buvimas ir žymenys, atitinkantys NMZL transformaciją į DLBCL. Šie rezultatai paryškina diagnostinius sunkumus, susijusius su NMZL, dėl specifinių imunohistocheminių žymenų stokos, ir pabrėžia histopatologinio vertinimo svarbą, siekiant atskirti NMZL nuo kitų limfomų, tokių kaip folikulinė limfoma. Šis atvejis akcentuoja ankstyvo nustatymo ir diferenciacijos svarbą limfomose, pasireiškiančiose pleuros išsiliejimu, kadangi transformacija į agresyvesnes formas, tokias kaip DLBCL, turi didelę įtaką prognozei ir gydymo taktikai. Nors transformacija diagnozės metu yra reta, gydytojai turėtų ją apsvarstyti NMZL atvejais su netipine klinikine išraiška.

## Introduction

Marginal zone lymphoma (MZL) comprises an indolent, uncommon subtype of B-cell non-Hodgkin lymphomas (NHL), derived from the marginal zone of the secondary lymphoid follicles [1]. The 5^th^ edition of the World Health Organization’s (WHO) Classification of Haematolymphoid Tumours has updated the categorization of Marginal Zone Lymphomas (MZL), now including four distinct entities: extranodal MZL of mucosa-associated lymphoid tissue (MALT), nodal MZL (NMZL), splenic MZL (SMZL), and pediatric nodal MZL (pNMZL) [2]. Primary cutaneous MZL (PCMZL) has been reclassified as a separate entity due to its unique clinicopathologic characteristics [2].

NMZL is a primary nodal B-cell neoplasm resembling splenic or extranodal (MALT) MZL but without splenic or extranodal disease involvement [2]. It accounts for 1–2% of lymphoid neoplasms and about 10% of MZL cases, making it the least common MZL subtype, with an incidence of 0.8 cases per 100,000 adults annually [3]. Typically diagnosed around age 60 with equal gender distribution, NMZL can also affect children, where it is classified as pediatric NMZL. It has been linked to autoimmune disorders, such as systemic lupus erythematosus and Sjögren’s syndrome, and potentially hepatitis C virus (HCV) [4].

Due to its rarity, reports on NMZL’s clinical profile are limited, but it often presents with asymptomatic or generalized lymphadenopathy, particularly in the cervical region. Bone marrow infiltration occurs in less than half of cases, with rare peripheral blood involvement. Pleural effusion at diagnosis correlates with a poorer prognosis, increasing relapse and reducing survival [5]. Extranodal disease is absent, and splenomegaly suggests SMZL. Lymphoma-related symptoms (B symptoms) occur in 10–20% of cases, and a serum monoclonal component is detected in about 10% of cases [5].

Diagnosis relies on clinical presentation, compatible histology, immunophenotype, and nodal involvement without extranodal or splenic disease. Differentiation from other indolent lymphomas, such as FL, requires careful evaluation of histopathology, cytogenetics, and immunophenotype [6]. NMZL cells typically express CD20, CD79a, PAX5, and BCL2 (43–100%), with negative CD5, CD23, and Cyclin D1; coexpression of CD43 and MNDA is common, whereas germinal center markers (e.g., CD10) are rare, distinguishing NMZL from FL [2,4]. Staging involves full-body imaging and bone marrow biopsy, with prognosis often being guided by the Follicular Lymphoma International Prognostic Index (FLIPI), which considers the age, nodal involvement, serum LDH, and hemoglobin levels [7,8]. Pleural effusion, found in about 20% of NHL, can complicate NMZL presentation. When present, it worsens prognosis and may cause respiratory distress, where thoracocentesis aids in both diagnosis and symptom relief [9]. Few cases of MZL presenting initially with pleural effusion have been documented [10,11]. A key concern for NMZL is histological transformation (HT) to diffuse large B-cell lymphoma (DLBCL), the most common NHL subtype [1].

HT, while rare in MZL, generally follows unclear mechanisms and occurs less often than FL [12,13]. HT is present in approximately 3% of NMZL [14]. Important numbers of centroblasts are frequently present in NMZL, which may lead to confusion with DLBCL. Most investigators accept the definition of HT as sheets of large cells arising from an indolent lymphoma with morphologic and immunophenotypic features suggestive of a high-grade lymphoma such as DLBCL, Hodgkin lymphoma, or Burkitt lymphoma [14]. Some investigators have also reported high proliferation (Ki-67) indices [15] and a higher proportion of centroblasts [16] as diagnostic criteria for HT. Clinical intuition and early recognition of an advanced stage disease and HT of NMZL are crucial, as these parameters contribute to morbidity and mortality, and the right choice of treatment is of great importance. In this case report, we present a rare, atypical NMZL case and attempt to answer some critical questions on the topic.

## Case presentation

A 67-year-old female presented to the emergency department complaining about a dry cough over the last month. The intensity of the cough increased during the night, without any accompanying symptoms. A chest X-ray, requested by a chest physician, revealed a medium-sized pleural effusion in the right half of the thoracic cage. The patient’s only preexisting medical condition was arterial hypertension, which was managed with losartan. The familial history was negative for tumors and chronic infectious disease.

The patient presented hemodynamically stable, with normal body temperature and normal respiratory capability [oxygen saturation (sO2): 96%, partial pressure of oxygen (pO2): 76 mmHg, fraction of inspired oxygen (FiO2): 21%]. Physical examination revealed no rash or cervical lymphadenopathy. Auscultation of the right base of the thoracic cage revealed decreased breath sounds. When the pleural effusion was aspirated, an exudate was found that met Light’s criteria: the ratio of pleural fluid protein to serum protein was close to 1, the ratio of pleural fluid LDH to serum LDH was close to 0.8, and the pleural fluid LDH was higher than two thirds of the upper limit of normal serum LDH. The cytologic testing of the fluid revealed a lymphocytic type, with 66% lymphocytes, 8% neutrophils, and 26% atypical cells.

**Figure 1:**
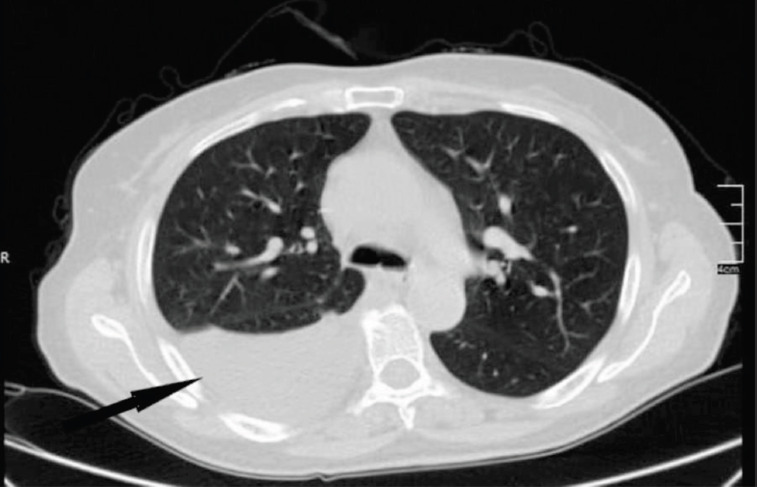
Chest computed tomography

During her hospitalization, a chest computed tomography (CT) scan with intravenous contrast revealed a significant right pleural effusion (Figure 1).

**Figure 2:**
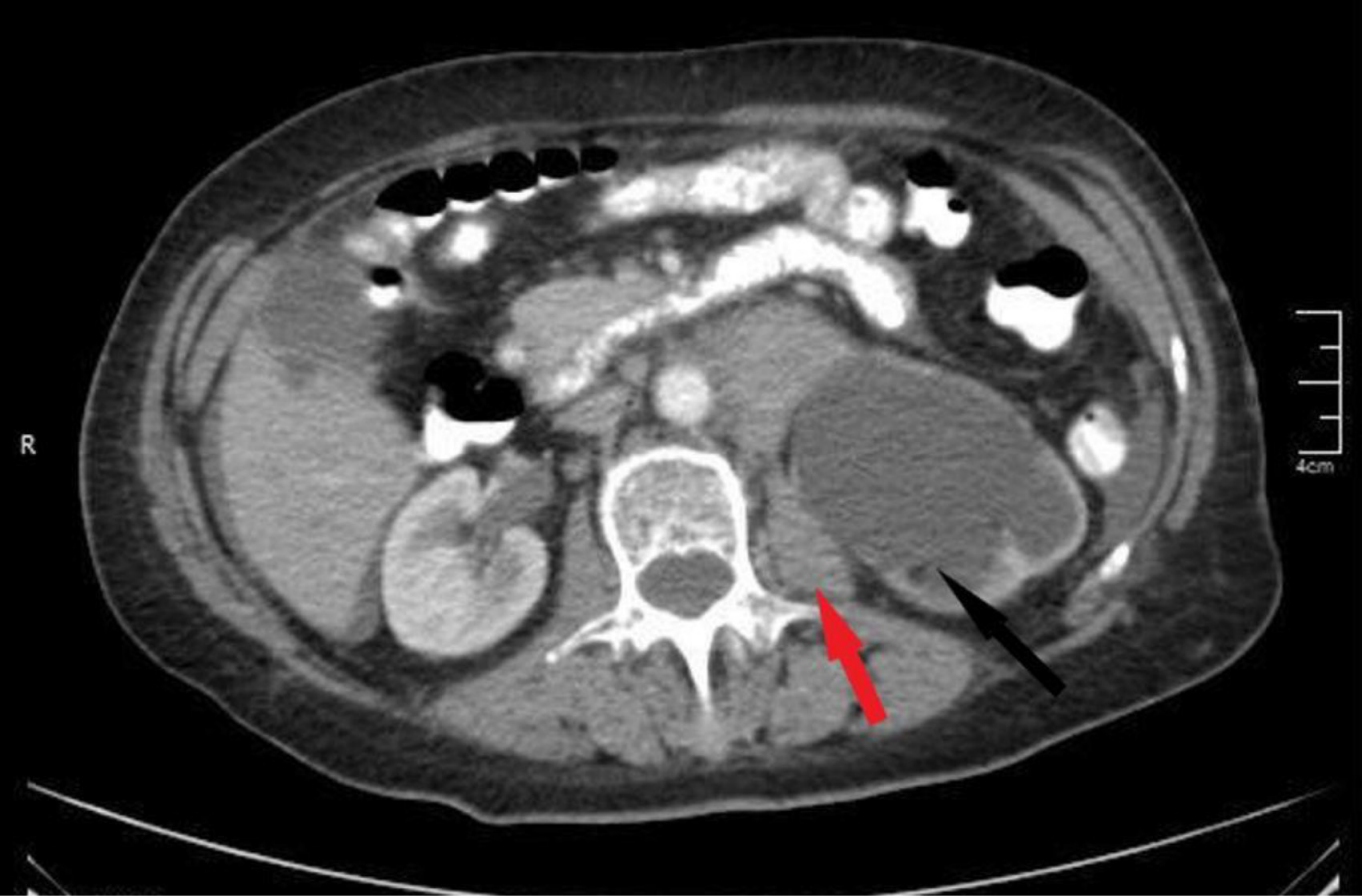
Computed tomography of the abdomen

An abdomen CT identified multiple enlarged individual lymph nodes, including one in the upper abdomen below the renal arteries (7.6 cm in diameter), another in the lower abdomen along the pelvic vessels (4.5 cm on the right side and 8.6 cm on the left side), and an inguinal lymph node on the left side measuring 5 cm in diameter. This lymphadenopathy also resulted in hydronephrosis in the left kidney (Figure 2).

The simple bronchoscopy demonstrated friable and hemorrhagic bronchial mucosa without any endobronchial mass. Heart ultrasound showed an ejection fraction of 60% without any signs of right heart strain, but only a medium level of tricuspid valve deficiency. The Mantoux skin test was negative, as were the B. Koch sputum cultures. Complete blood count, biochemical tests, immunological tests and serum protein electrophoresis were normal (Table 1).

**Table 1:** Serological and Immunological Test Results

Test Category	Tests Performed	Result
Viral Serologies	IgM for Epstein-Barr Virus, Cytomegalovirus, Toxoplasma	Negative
	HIV Test	Negative
	Anti-HCV, Hepatitis B Antibodies	Negative
Blood Cultures	Blind Blood Cultures	Negative
Rheumatologic Markers	Rheumatoid Factor	Normal
	Complement C3 and C4	Normal
Autoimmune Antibodies	Anti-CCP, Anti-dsDNA, Anti-Jo, Anti-RNP, ANA, Anti-Ro, Anti-La, Anti-Scleroderma (Anti-Scl)	Negative
Paraproteinemia Screening	Serum Protein Electrophoresis	Normal

anti-CCP: anti-Cyclic Citrullinated Peptide antibodies; anti-ds DNA: anti-Double-Stranded DNA antibodies; anti-Jo: anti-Histidyl-tRNA Synthetase antibodies; anti-RNP: anti-Ribonucleoprotein antibodies; ANA: antinuclear antibodies; antiscl: anti-Scleroderma antibodies; HIV: Human Immunodeficiency Virus; HCV: Hepatitis C Virus

**Figure 3:**
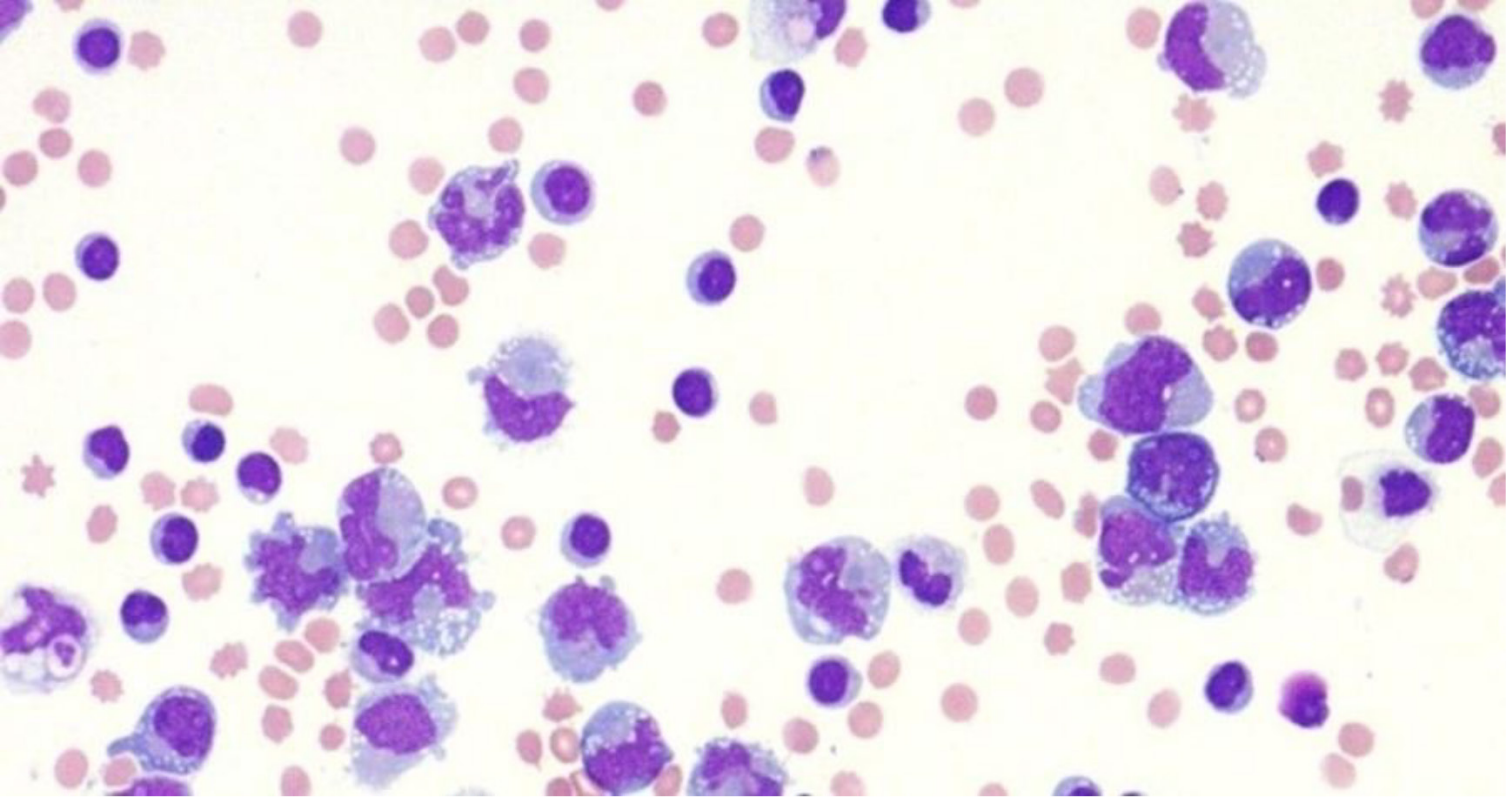
Pleural effusion cytology

Pleural effusion cytology revealed some large, atypical cells with poor connectivity and coarse chromatin aggregation (see Figure 3).

**Figure 4:**
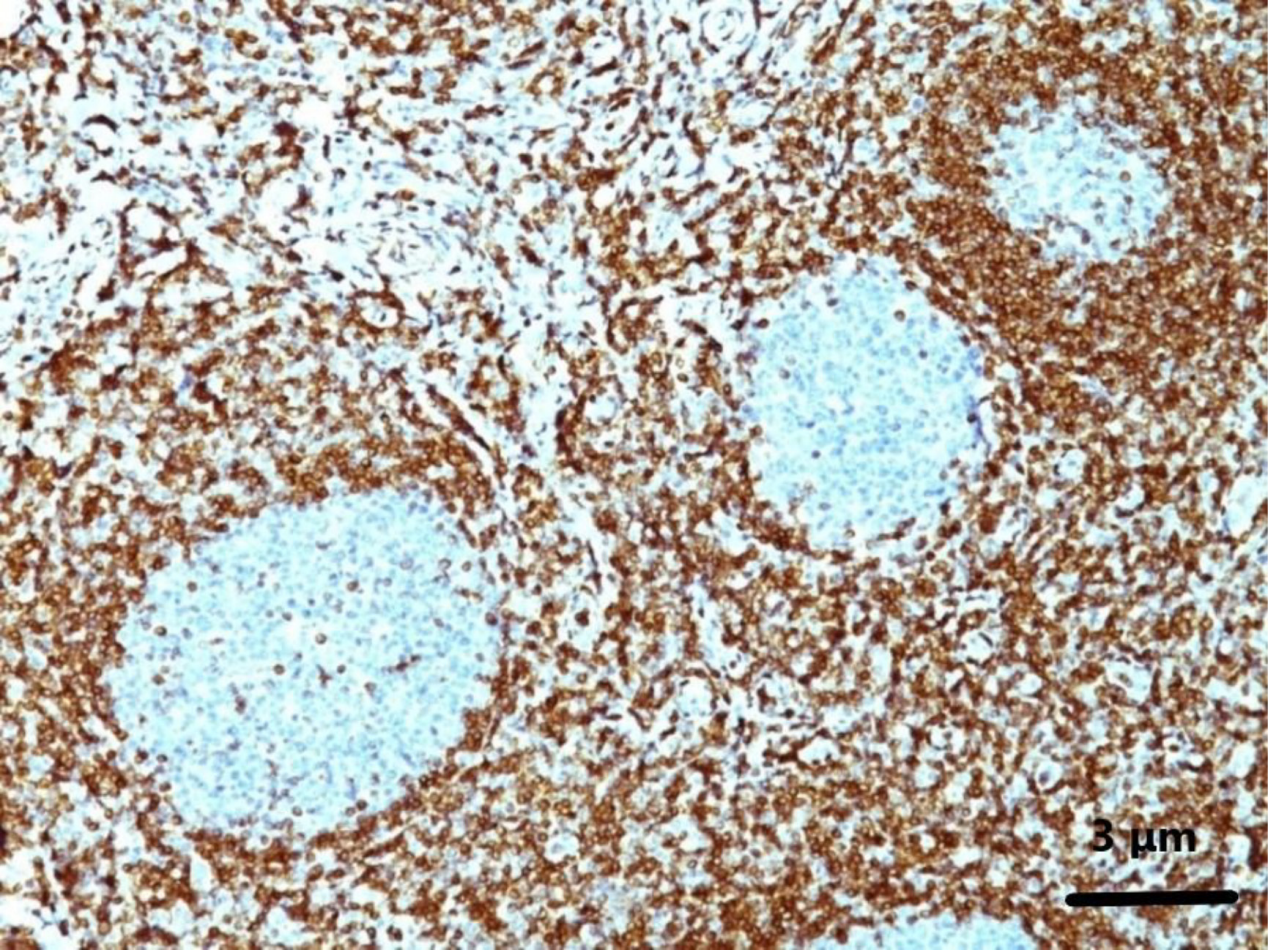
Immunohistochemical staining of lymph node biopsy specimen

The flow cytometry of the pleural fluid showed monoclonal B lymphocytes in 11.7% with CD10, CD38, and CD20 positive markers, while CD43 and CD5 were negative. The monoclonality of the B lymphocyte population in the pleural fluid was demonstrated through flow cytometric analysis, showing a significant restriction of the kappa-to-lambda light chain ratio, consistent with a monoclonal B-cell population. The gating strategy focused on identifying B cells using CD20 and CD19 markers, followed by the evaluation of light chain expression. Predominance of kappa-positive cells (or lambda-positive, depending on the findings) was observed, with a kappa-to-lambda ratio significantly deviating from the normal polyclonal range. Additionally, markers such as CD10 and CD38 further characterized the population, while the absence of CD5 and CD43 helped exclude other B-cell subtypes, thus supporting the diagnosis. No additional molecular testing, such as PCR for IGH gene rearrangements, was conducted as the light chain restriction was sufficient for confirming monoclonality in this context. According to these results, non-Hodgkin follicular lymphoma was suspected, and further investigation towards detection of BCL2 rearrangement was proposed (Figure 4).

**Figure 5:**
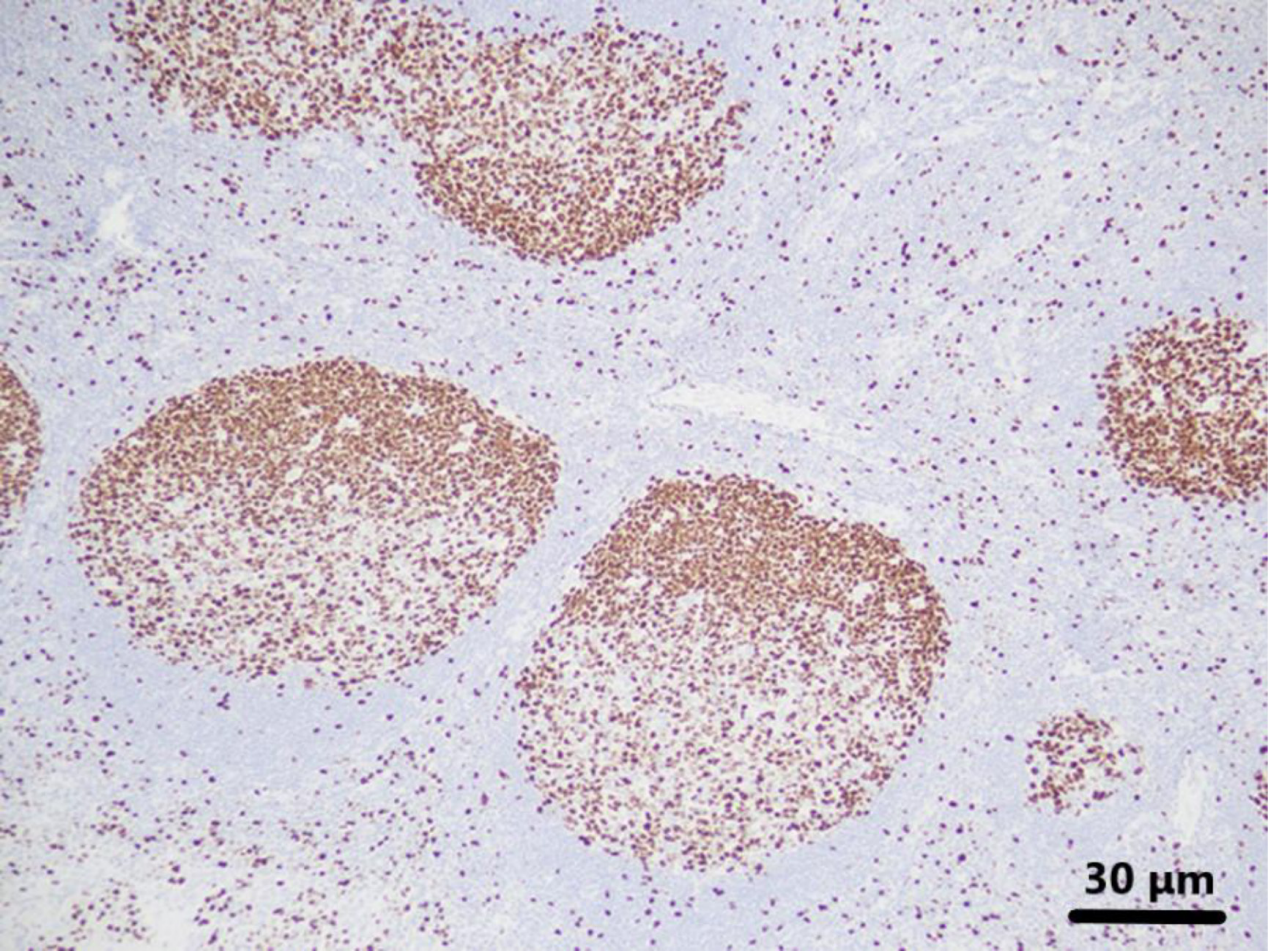
Histological examination of lymph node biopsy specimen

An en bloc biopsy of the left inguinal lymph node was performed, and histologic examination was suggestive of NMZL that in places appeared to have transformed into DLBCL with a high grade of malignancy. Some large B-cells were observed in the NMZL on histopathology. Also, an elevated Ki67 rate was detected (Figure 5).

We conducted several diagnostic and supportive tests to explore whether the pleural effusion was related to lymphoma or a reactive condition, as outlined in the case: a) Cytology and Immunohistochemistry: The pleural fluid showed monoclonal B lymphocytes, with markers CD10, CD38, and CD20 positive, but CD43 and CD5 negative. This indicated the possibility of an indolent lymphoma, potentially FL; b) Histological Examination: A biopsy from the left inguinal lymph node confirmed a diagnosis of NMZL, which had transformed into DLBCL in parts. This transformation suggested a high-grade malignancy. Immunohistochemical markers in the biopsy showed CD20, CD45, CD79a, and BCL2 positivity, while CD3, CD5, and CD10 were negative. BCL6 and Ki-67 were positive in areas with large cells; c) Bone Marrow Biopsy: The bone marrow examination revealed no lymphoma infiltration; and d) Cytogenetics and Additional Testing: While no molecular testing such as PCR for IGH gene rearrangements was done, the light chain restriction confirmed the monoclonality of the B cells, which was significant enough to support the lymphoma diagnosis.

Following the diagnosis, the patient was referred to the hematology department for the initiation of systemic treatment. Given the transformation of NMZL to DLBCL, the patient received standard first-line immunochemotherapy with R-CHOP (rituximab, cyclophosphamide, doxorubicin, vincristine, and prednisone) every 21 days. After six cycles, radiological assessment via PET-CT showed a complete metabolic response. The patient remained in clinical and radiological remission at the 12-month follow-up. No major adverse events were reported during the therapy, and the performance status improved significantly following the resolution of the pleural effusion. Maintenance therapy was not deemed necessary at that stage, and the patient continues under close hematological surveillance.

## Discussion

In this article, we present a rare case of NMZL with areas of transformation to DLBCL which initially presented as a pleural effusion. There are currently fewer than 10 published case reports of MZL presenting with pleural effusion [10,11,17,18,19]. When detecting pleural effusion, the differential diagnosis should encompass both transudative and exudative pleural effusion causes. Left ventricular failure, atelectasis, cirrhotic liver disease, hypoalbuminaemia, peritoneal dialysis, pulmonary embolism, nephrotic syndrome, or less commonly, constrictive pericarditis, hypothyroidism, malignancy (of which, up to 5% are transudates), Meigs’ syndrome, mitral stenosis, and urinothorax can cause transudative pleural effusions.

Exudative pleural effusions can be caused by an increase in capillary permeability and impaired pleural fluid resorption due to simple parapneumonic effusion, malignancy, or tuberculosis. Complex parapneumonic effusion, other infections, pneumonic embolism, autoimmune diseases, pancreatitis, oesophageal rupture, radiotherapy, asbestos, and drugs are less common causes. Pneumonia, congestive heart failure, and malignancy account for the vast majority of these cases [20]. The standard tests, initially proposed by Light et al. [21], distinguish transudates from exudates. NHL is responsible for up to 10% of malignant pleural effusions with a positive cytologic examination. The following mechanisms cause pleural effusions in NHL patients: (1) tumor infiltration into the pleura; (2) lymphatic obstruction from pulmonary and mediastinal lymph node infiltration; and (3) obstruction of the thoracic duct, leading to chylothorax [22]. In our case, right-side thoracentesis resulted in 66% lymphocytes. Light’s criteria confirmed the pleural fluid to be exudative. In order to examine hematological malignancies, we performed flow cytometry of pleural effusion and found proliferation of B lymphocytes with abnormal immune markers, suggestive of an indolent lymphoma, possibly FL. We should always include FL in the differential diagnosis of MZL and carefully exclude it due to areas of overlap. It is important at this point to comment on the significant challenge the diagnosis of MZL poses, as it lacks exclusive positive immunohistochemical and molecular markers. Luckily, in the case we present, histopathology of an excised enlarged left inguinal lymph node provided us with the diagnosis of a MZL with sites of transformation into DLBCL. The pleural effusion was considered a result of lymphatic obstruction due to infiltration of the pathological mediastinal lymph nodes detected. We categorized the pleural effusion as reactive and the MZL as nodal. HT of NMZL is an infrequent phenomenon. Different definitions have proposed vague criteria for this HT, with most authors citing sheets of large cells or a cluster of at least 20 large cells as the marker. Others define HT as cases where 30–50% of diffusely scattered large tumor cells are present [23]. Interestingly, our patient only reported cough as a symptom, while no B symptoms were present at the time of presentation, despite the histopathological presence of an aggressive lymphoma. Notably, our patient presented with a normal LDH level, whereas a raised LDH level is a common indicator of HT [24].

Given the lack of established properties, the clinicopathological characterization of the simultaneous secondary transformation of indolent lymphomas to DLBCL holds significant value. This characterization and the understanding of potential pathophysiological mechanisms leading to HT will enable the use of appropriate treatment strategies, primarily immunotherapy as the first-line treatment for aggressive lymphoma-type lymphomas.

## Conclusions

This is a rare case of pleural effusion detected concurrently with NMZL with areas of DLBCL transformation. Timely diagnosis is substantial for increasing the survival rates. The significance of differential diagnosis for pleural effusions should be remembered by clinicians. In cases of MZL where specific markers are absent, histopathological characterization of a lymph node is critical and should always be required, as results obtained through cytometry may be unclear or unreliable.
